# The Effect of Autonomy and Self-Control on Changes in Healthy Lifestyles of Inactive College Students through Regular Exercise

**DOI:** 10.3390/ijerph191710727

**Published:** 2022-08-28

**Authors:** Jihoon Ahn, Inwoo Kim

**Affiliations:** 1 Department of Physical Education, Seoul National University, Seoul 08826, Korea; 2 Department of Sports Culture, Dongguk University, Seoul 04620, Korea

**Keywords:** autonomy, self-control, healthy lifestyles, regular exercise, university students

## Abstract

This study aimed to verify the influence of autonomy and self-control as psychological factors on the changes in lifestyles of inactive college students by participating in regular exercise. A total of 188 university students in Seoul, Korea, taking physical fitness classes for 5 weeks held three times a week participated in the surveys. Surveys were conducted in the first session (T1) and 15th session (T2) of the classes. Autonomy in exercise participation and self-control were measured at T1, and healthy lifestyle was measured at both T1 and T2. A paired *t*-test was used to measure the changes in healthy lifestyle between two time points, and hierarchical regression analysis was conducted to determine the effect of autonomy in exercise participation and self-control measured at T1 on the healthy lifestyle score at T2. According to the analysis, participants’ healthy lifestyles were improved with a statistically significant difference between pre- and post-exercise. Furthermore, the levels of autonomy and self-control before the fitness classes positively influenced the participants’ healthy lifestyle after the classes even when the influence of healthy lifestyle measured before the classes was controlled. Thus, it was confirmed that autonomy for participation and self-control are important to change one’s healthy lifestyle through regular exercise participation.

## 1. Introduction

“Lifestyle” may be defined differently by researchers, but the most widely accepted definition is “a set of habits of an individual” [1]. Recently, in the field of health science, there has been an increased interest in lifestyle as a game changer to understand health and quality of life in modern society because health has become a key value of lifestyle [2]. Various factors such as participation in exercise and physical activities, relaxation and leisure, diet, and psychological stability are required for a healthy lifestyle, but some researchers considered physical activity as the most important factor [3]. They explained that an increase in physical activity could lead to changes in other lifestyle factors.

Although a healthy lifestyle is important for quality of life and physical activity is a key element of the lifestyle, the exercise frequency and duration of those in their 20s fall short of the average for all ages [4]. Young adults face various stressors as they experience the transition from adolescence to adulthood, and this may lead to their unhealthy lifestyle, including bad eating habits and lack of exercise [5]. Healthy lifestyles formed during this period have a significant impact on their lifelong health, and it is important to promote regular exercise participation in young people to create healthy and active lifestyles [6]. Regular exercise performs a key function of changing an individual’s lifestyle by cultivating a positive attitude and confidence toward a healthy life along with an increase in physical activity [7]. Therefore, it is necessary to pay much attention to promoting regular exercise participation of young people [5].

Emphasizing the benefits of participating in exercise is not enough to promote people to engage in regular exercise and form a healthy lifestyle. Most people, even those who do not exercise, know that participating in exercise is beneficial in improving their physical, mental, and social health, but still many people fail to engage in regular exercise. To change the behavior of these people, not only the environmental factors but also the psychological factors are important [8]. Ryan, Lynch, Vansteenkiste, and Deci [9] claimed that autonomy in certain actions is the basis for individual behavior patterns and thoughts to change.

Autonomy refers to the characteristic of trying to take part in a particular action according to one’s own principles and choices [10]. Physical and psychological well-being can be directly promoted when autonomy is assumed [11]. Moreover, autonomy is an important factor in psychological growth and development and is essential to identify environmental factors that support or hinder one’s development [12]. To form a healthy behavior pattern or a healthy lifestyle, an individual is faced with a situation that requires constant motivation [13]. In other worlds, when people aim to continue health behaviors such as participating in exercise, consuming healthy food, and cutting down smoking and drinking, a lack of autonomy for such behaviors makes it difficult to maintain motivation [14]. Thus, it is believed that the autonomy to participate in regular exercise plays an important role in creating a healthy lifestyle for students [15].

For everyday behavior patterns to change to the desirable form, people must control themselves to overcome temptation and impulse [16]. Continued exposure to tempting situations depletes an individual’s self-control and makes it difficult to continue ideal goal behavior [17]. Thus, the total amount of personal self-control resources is a major psychological factor required for lifestyle changes along with autonomy.

Self-control is defined as the ability to not only curb unwanted behavioral tendencies on one’s own, but also change one’s behavior patterns [18]. This ability has been shown to function positively in areas such as academic achievement, diet, drugs, and smoking because they intentionally suppress behaviors one deems inappropriate by oneself to achieve greater satisfaction and results [19]. Self-control, as the ability to replace current emotions and habitual behaviors with desirable ones through conscious effort, serves as a very useful tool in life [20].

Individuals with good self-control can lead a healthy and satisfying life by successfully achieving their goals in their daily lives [21]. Self-control acts as an important psychological factor that moderates various health behaviors, including exercise [20]. People who lack self-control can act impulsively and make immature decisions when faced with physical and mental stress [22] and experience failure in changing their behavior to the goal behavior pattern [23]. This can be interpreted to mean that university students can overcome various factors that hinder their healthy lifestyle by developing self-control.

Previous research on lifestyles focused on analyzing the state of lifestyles or on verifying the relationship between leisure and consumption patterns, and psychological variables such as happiness, satisfaction, and self-esteem [24,25,26]. Many studies analyzed lifestyle only by measuring physical strength and physical activity because they could not use other appropriate measures owing to the wide range of lifestyle concepts. Cale and Harris [27] and Green [7] provided results from interviews that various physical activities experienced through exercise and sport can change one’s lifestyle by improving one’s positive attitude and self-confidence in daily life, but they did not perform empirical and experience-based analyses.

In this study, we tried to focus on the changes in lifestyle that accompany participation in regular exercise and to identify psychological factors that could predict the changes in lifestyle. In addition, we tried to examine lifestyle with a measure that could encompass physical activity, psychological health, and healthy habits in daily life to explain a lifestyle more broadly.

In summary, this study aimed to verify the impact of autonomy and self-control on changes in the healthy lifestyles after participating in regular exercise in university students who did not exercise. Thus, the main purpose of the present study was to provide useful implications that can help young adults who are not able to properly take care of their health due to various stresses to form healthy lifestyles.

## 2. Methods

### 2.1. Participants

The participants of this study were university students who were taking physical fitness classes from a university located in Seoul, Korea. Using the convenient sampling method, the participants were sampled only if the students who did not exercise at all outside of class participated in the survey. After recruiting 207 students, 19 students who canceled the class or provided an incomplete questionnaire were excluded to yield a final sample of 188 participants. There were 93 men (49.5%) and 95 women (50.5%), and the mean age of the participants was 20.76 years (standard deviation (*SD*) = 1.955). It was ensured that the participants did not have experience in regular participation in exercises, to analyze the lifestyle changes in university students with insufficient physical activity.

### 2.2. Study Procedure

Among the students who voluntarily enrolled in the physical fitness classes, the students who did not exercise outside of the class were encouraged to participate in the study. The physical fitness classes were held three 100 min sessions per week for 5 weeks. All the classes consisted of the same contents and were taught by same instructor, and there were no differences according to gender or the students’ preference. One session consisted of approximately 30 min of moderate-intensity aerobic exercise and approximately 70 min of strength training through weightlifting, including appropriate rest periods for beginners. In addition, a survey was conducted only for students who had never participated in regular exercise. Prior to conducting the survey, permissions were obtained from the instructor and the internal review board of the researchers’ university, and the questionnaire was distributed only to students who voluntarily agreed to participate in the survey. The survey took approximately 10–15 min to complete, and the completed surveys were directly collected on site by the researcher. Surveys were conducted in the first session (T1) and 15th session (T2) of the fitness classes. Autonomy in exercise participation and self-control were measured at baseline T1, and healthy lifestyle was measured at both T1 and T2 points to examine the change before and after. Participants were asked to enter the last four digits of their phone numbers on their survey sheets at both time points and these numbers were used as the code to collect pretest-posttest data.

### 2.3. Measures

Participants’ gender and age were set as background variables, and the following instruments were used to measure autonomy in exercise participation, self-control, and healthy lifestyles.

#### 2.3.1. Autonomy in Exercise Participation

To measure autonomy in exercise participation, the Behavioral Regulation in Exercise Questionnaire-2 (BREQ-2; [28]) was used. BREQ-2 is comprised of amotivation (4 items), external regulation (4 items), introjected regulation (3 items), identified regulation (4 items), and intrinsic regulation (4 items) as subscales (19 items in total), and is rated on a 5-point scale ranging from 1 (not true for me) to 5 (very true for me). Examples of included items are “I don’t know why I have to exercise regularly” (amotivation factor), “I exercise because others demands it” (external regulation factor), “I feel guilty if I do not exercise” (introjected regulation factor), “I exercise because I think that it is important to exercise” (identified regulation factor), and “I exercise because exercising is fun” (intrinsic regulation factor). A confirmatory factor analysis to test the construct validity of the scale showed that the goodness of fit index of the measurement model was χ^2^ = 376.509, degrees of freedom (df) = 142, and the null hypothesis was rejected (*p* < 0.001). The results also yielded the following values: Tucker Lewis index (TLI) = 0.945, comparative fit index (CFI) = 0.957, root mean square error of approximation (RMSEA) = 0.073, and the validity of the construct model was verified. Furthermore, the reliability analysis showed that the internal consistency of each factor was as follows: amotivation 0.900, external regulation 0.932, introjected regulation 0.894, identified regulation 0.943, and intrinsic regulation 0.876. To identify the autonomy in participation in regular exercise using this scale, the relative autonomy index (RAI) was calculated. Among various methods to calculate RAI, the Markland [29] equation, which has been shown to have the highest validity and reliability, was used [30]. This equation puts a different weight on each sub-factor depending on the degree of autonomy [29]: [(amotivation × −3) + (external regulation × −2) + (introjected regulation × −1) + (identified regulation × 2) + (internal regulation × 3)]. According to the equation, a higher RAI value indicates a higher degree of autonomy.

#### 2.3.2. Self-Control

To measure self-control, Cho and Kwon’s [31] revision of the self-control scale (SCS) developed by Tangney and colleagues [18] was used. SCS was comprised of 5 factors (restraint and self-discipline, deliberate/non-impulsive act, healthy habits, work ethic, and reliability) and 36 items, but this was restructured through research with Korean university students by Cho and Kwon [31]. Three factors found to indicate the best goodness of fit of the model (healthy habits, restraint and self-discipline, and non-impulsivity) and 26 items were used in the revised scale. Each item was rated on a 5-point scale ranging from 1 (not at all like me) to 5 (very much like me). Examples of items are “I have a hard time breaking bad habit” (healthy habits factor), “I can act effectively to achieve a long-term goal” (restraint and self-discipline factor), and “There are times when I ruin things in the spur of the moment” (non-impulsivity factor). A confirmatory factor analysis was performed to verify the construct validity of the scale. One item on the restraint and self-discipline factor and two items on the non-impulsivity factor that had a factor loading value lower than 0.50 were deleted. Finally, a secondary confirmatory factor analysis was performed on 23 questions across 3 factors. The results showed that a goodness of fit index of χ^2^ = 318.664, df = 227, and the null hypothesis was rejected (*p* < 0.001). The results also yielded the following values: TLI = 0.908, CFI = 0.911, RMSEA = 0.085, and the validity of the construct model was verified. Furthermore, the reliability analysis showed that the internal consistency of each factor was suitable at 0.842 for healthy habits, 0.875 for restraint and self-discipline, and 0.895 for non-impulsivity. Self-control was calculated as the sum of the scores of the 23 items divided by the number of items. Greater calculated values indicated better ability to self-control.

#### 2.3.3. Healthy Lifestyles

To measure changes in the healthy lifestyle after regular exercise, the healthy lifestyles belief scale (HLBS) developed by Melnyk and Small [32] was used. The original scale is comprised of 16 items across 2 factors: beliefs about physical activity (8 items) and healthy lifestyle habit and psychological health (8 items) [33]. However, because the goodness of fit of the model comprised of two factors was not good, exploratory factor analysis, confirmatory factor analysis, and reliability analysis were performed to test the factor structure and reliability. First, to determine the appropriate number of factors, factors with eigen values that exceeded 1.0 were derived using the maximum likelihood. As a result, the number of factors was 3. Three cross-loaded items with factor loads that did not exceed 0.30 were deleted and exploratory factor analysis was performed on 3 factors and 13 items. The Kaiser–Meyer–Olkin fit index was 0.81, indicating that the correlation among items was good [34]. All factor loads also exceeded 0.40, showing good values. Based on the results of the exploratory factor analysis, confirmatory factor analysis was performed on the sample of 188 participants. The results showed a goodness of fit index of χ^2^ = 119.207, df = 62, and the null hypothesis was rejected; the results showed TLI = 0.914, CFI = 0.927, RMSEA = 0.073, and the validity of the model was verified. Through the factor analyses, HLBS was restructured to the following three factors: beliefs about physical activity, beliefs about healthy lifestyle habits, and beliefs about psychological health. The reliability analysis showed that the internal consistencies of the factors were good at 0.919 for physical activity, 0.936 for healthy lifestyle habits, and 0.886 for psychological health. Specific items included “I think I will feel better if I exercise regularly” (beliefs about physical activity), “I can regularly consume healthy food” (beliefs about healthy lifestyle habits), and “I can resolve stress from others in a positive way” (beliefs about psychological health). Each item was rated on a 5-point scale from 1 (not true at all) to 5 (very true). The healthy lifestyle score was calculated through a sum of the scores of the 13 items and dividing that number by the number of items. Greater calculated values indicated healthier lifestyles.

### 2.4. Analysis

The data were analyzed using AMOS 21.0 and SPSS 26.0 statistical programs. First, descriptive statistical analyses and correlational analyses were performed to identify the general trends and characteristics of the data (such as mean and standard deviation). Next, factor analyses were performed to verify the construct validity of the surveys, and reliability analysis was performed for each factor (Cronbach’s *α*). Furthermore, to measure changes in the participants’ healthy lifestyle, a paired *t*-test was performed. To test whether autonomy in exercise participation and self-control had a significant impact on posttest healthy lifestyle (T2) even after controlling for the impact of pretest healthy lifestyle (T1) on posttest healthy lifestyle, hierarchical regression analyses were performed.

## 3. Results

### 3.1. Measured Variables: Descriptive Statistics and Correlation

The results of the descriptive statistical analyses and correlational analyses to identify the general trends and correlations of major variables are shown in Table 1.

All variables’ skewness (≤2) and kurtosis (≤4) satisfied the standard value [35], showing that the measurements were normally distributed. The RAI, which represents the participants’ autonomy in exercise participation, was calculated using Markland’s [28] equation. The mean RAI (T1) was 10.85 (*SD* = 4.786). Furthermore, the mean self-control (T1) of the participants was 3.37 (*SD* = 0.455), the healthy lifestyle (T1) was 3.23 (*SD* = 0.347), and the healthy lifestyle (T2) was 3.51 (*SD* = 0.354).

The results of examining the correlation among the major variables showed that the healthy lifestyle (T2) had a statistically significantly positive correlation with the healthy lifestyle score (T1), self-control (T1), and the RAI (T1). Specifically, healthy lifestyle (T1) had a high correlation (*r* = 0.812, *p* < 0.01) with healthy lifestyle (T2), and the correlation values for the RAI (T1) and self-control (T1) with healthy lifestyle (T2) were 0.293 (*p* < 0.01) and 0.356 (*p* < 0.01), respectively. Self-control (T1) and the RAI (T1) also showed positive correlations with pretest healthy lifestyle (T1), at 0.168 (*p* < 0.05) and 0.184 (*p* < 0.05), respectively. Meanwhile, the RAI (T1) and self-control (T1) were not significantly correlated with each other. Furthermore, gender and age, which were investigated as background variables, were also not found to be significantly related to other variables.

### 3.2. Changes in Healthy Lifestyle of Participants after Regular Exercise

To assess changes in the healthy lifestyle after participating in regular exercise, a paired t-test was performed. Differences in the healthy lifestyle scores between T1 and T2 are shown in Table 2. The mean posttest healthy lifestyle score was 3.51, which was a 0.28 increase from the pretest score. The two means were found to show a statistically significant difference (*t* = −17.848, *p* < 0.001).

### 3.3. Impact of Autonomy (RAI) and Self-Control on Changes in Healthy Lifestyle

To test the impact of participants’ autonomy (T1) and self-control (T1) on changes in their healthy lifestyles, a hierarchical regression analysis was performed. Considering the high correlation (*r* = 0.812) of the pretest healthy lifestyle (T1) with the posttest healthy lifestyle (T2), it was tested whether the RAI (T1) and self-control (T1) had independent effects after controlling for pretest healthy lifestyles (T1). Accordingly, the posttest healthy lifestyle (T2) was set as the dependent variable. The pretest healthy lifestyle (T1) was entered as the independent variable in the first step, and the RAI (T1) and self-control (T1) were stepwise entered in the second step to perform the regression analysis.

To test the multicollinearity of this hierarchical regression analysis, the variance inflation factor (VIF) and tolerance (TOL) were assessed as test statistics. The VIF value was under 10 (1.007~1.012) and the TOL value was over 0.1 (0.988~0.993), both satisfying the standard values. Thus, it was judged that there was no risk of multicollinearity. Moreover, the Durbin–Watson value was found to be 1.846, which is close to the standard of 2. Thus, the possibility of autocorrelation was excluded, and the conditions of independence were met [36].

As shown in Table 3, the F values of Model 1 and Model 2 were found to be statistically significant, showing that the regression model was good. The explanatory power of the pretest healthy lifestyle (T1) on the posttest healthy lifestyle (T2) in Model 1 was 65.7% (*F* = 361.563, *p* < 0.001) and significant. The explanatory power of Model 2, to which the RAI (T1) and self-control (T1) were added, was 80.3% (*F* = 428.830, *p* < 0.001), or an increase of 14.4%. When the relative influences of the RAI (T1) and self-control (T1) were compared, it was found that the impact of self-control (*β* = 0.296, *p* < 0.001) on the posttest healthy lifestyle (T2) was greater than the impact of the RAI (*β* = 0.214, *p* < 0.001).

## 4. Discussion

For those in their 20s who have just entered adulthood, regular exercise at this time has the function of improving health and laying the foundation for lifelong sports development [37]. Considering that an increase in physical activity is directly linked to an active and healthy lifestyle [3], regular exercise plays an important role of establishing a foundation of health management. This study identified the changes in university students’ healthy lifestyle through participation in regular exercise and verified that autonomy in exercise participation and self-control were important psychological variables that could predict the changes in a healthy lifestyle.

The RAI and self-control were shown to have significantly positive correlations with the posttest healthy lifestyle (T2). This means that students who voluntarily chose to participate in exercise and students who could exert self-control in their daily lives could form a positive lifestyle through regular exercise. These results support those of Jang [38], who found that one can better overcome obstacles to change one’s behavior in the presence of the initial autonomy. Meanwhile, self-control is an individual’s energy resource that is used in all aspects of daily life; when attempting to change to a particular behavioral pattern, self-control repeatedly undergoes consumption, depletion, and recovery when one faces a situation that requires control [17]. Therefore, it can be predicted that when a health behavior goal is set, the changes in lifestyle would be larger in students with greater self-control.

This study also found that the healthy lifestyle score increased from pretest (T1) to posttest (T2) with statistical significance. A healthy lifestyle, as measured in this study, was a concept that included psychological stability and healthy lifestyle habits. Therefore, the changes found through the t-test can support the research by Nuviala and colleagues [3], that regular physical exercise can lead to changes in the overall lifestyle. These results are in line with previous findings that individuals who engage in regular exercise have more healthy dietary habits [39] and experience psychological well-being through the enhancement of self-esteem satisfaction [40]. However, regular exercise is difficult to maintain without the strong will of the individual. Fishbein and Ajzen [41] emphasized psychological and mental factors to promote changes in behavior by stating that individuals’ psychological characteristics lead to dynamic and active participation in health behaviors.

Therefore, this study aimed to test the effects of autonomy and self-control (as psychological factors) on the changes in the healthy lifestyle of students participating in regular exercise. Because the correlation between pretest (T1) and posttest (T2) healthy lifestyle scores was high at 0.812, hierarchical regression analyses were performed to control the effect of the pretest score (T1). As discussed earlier, the explanatory power increased when autonomy and self-control were additionally entered (Δ*R*^2^ = 0.144) to the model with the pretest healthy lifestyle as the independent variable, compared to the previous model (*R*^2^ = 0.657). This can be interpreted to mean that autonomy and self-control have independent effects on the pretest–posttest changes even after controlling for the pretest healthy lifestyle score.

Considering that changing behavior patterns is not an easy task, it is a noteworthy finding that autonomy in exercise participation and self-control can promote changes in lifestyle. These results support the finding that an individual’s level of autonomy in the change to a particular behavior pattern determines the continuation of this behavior [42]. It also in line with the claim that autonomy is needed to change individual behavior and thinking [43]. Furthermore, Lopez, Milyavskaya, Hofmann, and Heatherton [44] found that a high level of autonomy for a specific behavior reduces the negative emotions that induced the behavior, allowing for a long-lasting habit of behavior. This finding is helpful to understand that students who voluntarily participate in exercise can make a change to a positive and healthy lifestyle.

Meanwhile, the results of examining the relative impact (*β*) of the factors on the posttest healthy lifestyle showed that self-control had a greater effect than autonomy. Self-control has been shown to have a positive effect on drug abuse [45] and alcohol [46]. Lower self-control is associated with vulnerability with repeated suppression attempts, such as diets [47]. Based on previous findings and the results of this study, self-control was understood to be an important variable that could control unhealthy behavior [48]. A common research finding on self-control is that people with greater self-control traits can suppress impulsive behavior when exposed to situations that require control. It is difficult to change behavioral habits and mindsets in daily life even while pursuing health behavior, because people are constantly faced with situations requiring control [16]. Through this study, it was verified that self-control can function as a useful tool that can replace an individual’s habitual behaviors with desirable behaviors [20].

Based on the limitations of this study, the following suggestions are made. First, since this study was conducted with students taking a course called “physical fitness” by the same instructor, it is difficult to generalize to other exercise participants. A research design that selects subjects who participate in various exercise or sports events is essential for the wide application of the research results. Second, the healthy lifestyle scale used in this study measures beliefs in pursuing a healthy lifestyle. Considering that beliefs and intentions do not necessarily lead to action [49], more reliable results could be obtained if a scale that measures actual changes in lifestyle could be developed. Third, it is difficult to explain whether the changed lifestyle was maintained, because measurements were taken only twice—before and after exercise participation. A wide range of research would be possible if lifestyle changes are checked through follow-up tests following posttest.

## 5. Conclusions

Through this study, the influence of autonomy and self-control as psychological variables that can contribute to the formation of healthy living habits was identified. Therefore, if autonomy in exercise participation and self-control can be fostered, the goal of exercise to improve quality of life through physical activity can be actualized. To seek a change to a healthy lifestyle, regular exercise must be involved rather than one-time physical activity [3], along with cultivating the ability of self-control and providing motivation to participate in regular exercise autonomously.

## Figures and Tables

**Table 1:** Descriptive statistics and Pearson’s correlations of measured variables.

	*M*	*SD*	Skewness	Kurtosis	A	B	C	D
A	10.85	4.786	−1.265	2.263	1			
B	3.37	0.455	0.013	−0.987	0.087	1		
C	3.23	0.347	0.228	0.141	0.168 *	0.184 *	1	
D	3.51	0.354	0.258	−0.028	0.293 **	0.356 **	0.812 **	1

Note: * *p* < 0.05, ** *p* < 0.01; A, relative autonomy index (RAI) (T1); B, self-control (T1); C, healthy lifestyle (T1); and D, healthy lifestyle (T2).

**Table 2:** Comparison of healthy lifestyle scores before and after participation in regular exercise.

Categorization	*M*	*SD*	*t*
Pretest (T1)	3.23	0.347	−17.848 ***
Posttest (T2)	3.51	0.354	

Note: *** *p* < 0.001.

**Table 3:** Hierarchical regression analysis showing impact of autonomy and self-control on changes in healthy lifestyle.

Independent Variables		*β*	*t*	*R* ^2^	Δ*R*^2^	*F*
Dependent Variable = Healthy lifestyle (T2)						
Model 1	Healthy lifestyle (T1)	0.812	19.015 ***	0.657	-	361.563 ***
Model 2	Healthy lifestyle (T1)	0.781	23.837 ***	0.803	0.144	428.830 ***
	RAI (T1)	0.214	6.528 ***			
	Self-control (T1)	0.296	9.011 ***			

Note: *** *p* < 0.001, RAI: relative autonomy index.

## Data Availability

The data presented in this study are available on request from the corresponding author.
